# Role of autoantibodies in the pathophysiology of irritable bowel syndrome: a review

**DOI:** 10.3389/fphys.2024.1359003

**Published:** 2024-03-05

**Authors:** Yu Zhang, Jiazhi Liao, Wenjuan Fan

**Affiliations:** Department of Gastroenterology, Tongji Hospital, Tongji Medical College, Huazhong University of Science and Technology, Wuhan, Hubei Province, China

**Keywords:** pathogenesis, diagnosis, autoantibody, autoimmune gastrointestinal dysmotility, irritable bowel syndrome

## Abstract

Irritable bowel syndrome (IBS) is a chronic, recurrent disorder that is characterized by abdominal pain associated with defecation. IBS was previously considered to manifest without any structural alterations until the discovery of post-infection IBS. An increasing body of published evidence indicates that immune activation plays an important role in the development of IBS. Nevertheless, the pathophysiology of IBS, including mainly visceral hypersensitivity and gastrointestinal dysmotility, has not yet been explicitly elucidated. The observation of potential inflammatory degenerative neuropathy, including neuronal degeneration, spearheaded research on autoimmune responses targeting the enteric nervous system. Subsequently, several autoantibodies were detected in the sera of IBS patients, among which some were presumed to exert a pathogenic influence or be associated with the etiology of gastrointestinal dysmotility in IBS. Moreover, certain specific autoantibodies evidently served as biomarkers to facilitate the differentiation between IBS and other related diseases. Therefore, we aimed to present an overview of autoantibodies reported in the sera of IBS patients and highlight their significance in diagnosing and comprehending the pathophysiology of IBS. Consequently, we propose a therapeutic strategy from an autoimmune perspective.

## 1 Introduction

Irritable bowel syndrome (IBS) is a chronic disorder of gut-brain interaction (DGBI) characterized by altered bowel habits and recurrent abdominal pain. As evidenced by the Internet survey, the prevalence of IBS ranges from 4.1% to 10.1% in the global population based on Rome IV and Rome III criteria (Sperber et al., 2021). Although IBS does not impact the life expectancy of patients, the associated impairment in health-related quality of life is substantially worse than that observed in organic diseases, such as chronic liver disease or inflammatory bowel disease (IBD) (Younossi et al., 2001; Blagden et al., 2015). IBS accounts for approximately 12% of primary care visits and 28% of gastroenterology referrals in the United States (Everhart and Ruhl, 2009) and may account for up to 3.3% of the total healthcare budget in China (Zhang et al., 2016). IBS poses a concerning challenge to patients and doctors as the therapeutic approaches are conventionally targeted at the predominant or most critical symptoms rather than addressing the underlying pathophysiology. Approximately 43.2% of IBS patients express contentment following the alleviation of symptoms (Fan et al., 2017), and approximately one-third of primary care physicians and gastroenterologists concur that IBS management is highly frustrating (Törnblom et al., 2018). IBS is one of the most widely recognized DGBIs and reportedly manifests without any explicit pathological alterations. Despite substantial advancements in the diagnosis and treatment of IBS, there remain numerous unresolved issues with multifactorial aspects, particularly regarding its etiology.

Following acute gastroenteritis, a subset of patients experience IBS symptoms, which is referred to as post-infection IBS (PI-IBS). PI-IBS is an optimal natural model, indicating that a specific group of patients may suffer from IBS owing to an underlying organic basis (Ghoshal, 2022). Previously, researchers primarily concentrated on abnormalities in the motility of the gastrointestinal tract, visceral sensation, brain-gut interaction, and psychophysical stress; however, these aberrations could partially account for the pathogenesis of IBS (Chey et al., 2015). During the last 2 decades, a growing body of evidence has revealed that immune activation plays a vital role in the pathophysiology of IBS. The colonic area of the lamina propria, occupied by total immune cells, CD3^+^, CD4^+^, and CD8^+^ T cells, and mast cells, is considerably larger in IBS patients than in healthy individuals (Cremon et al., 2009). IBS patients exhibited an augmented cellular immune response, with elevated production of proinflammatory cytokines, including TNF-α, IL-1β, and IL-6 (Liebregts et al., 2007). Studies investigating humoral immunity have reported that IBS patients predominantly presenting with diarrhea (IBS-D) displayed an increased proliferation and activation of B lymphocytes and immunoglobulin (Ig) production in mucosal jejunal biopsies compared with those in healthy individuals (Vicario et al., 2015). Compared with healthy individuals, IBS-D patients presented with differential expression of humoral pathways and enhanced activation and proximity of plasma cells to nerves and IgG concentrations, portraying humoral activity as a pivotal contributor to gut dysfunction and clinical symptoms (Pardo-Camacho et al., 2022).

In 2002, a prominent study involving full-thickness biopsy of the jejunum of IBS patients revealed lymphocyte infiltration into the myenteric plexus and indications of neuron degeneration, namely, cytoplasmic swelling, vacuolization, and shrunken or ruptured nuclei (Törnblom et al., 2002). Dr. Wood reported that patients with inflammatory degenerative neuropathy might have transition from IBS-like symptoms to a state of chronic pseudo-obstruction, indicating that the etiology of IBS may be comparable with that of inflammatory degenerative neuropathy (Wood, 2000). Research examining the prevalence of autoantibodies in IBS patients was triggered by the discovery of anti-neuronal nuclear antibodies, also known as anti-Hu antibodies, in paraneoplastic syndrome (PNS) patients with gastrointestinal complications (De Giorgio et al., 2003). Nevertheless, in certain rare cases, they manifest without the presence of distant tumors. Additionally, the emergence of a novel clinical entity designated autoimmune gastrointestinal dysmotility, has been documented, which represents a type of autoimmune dysautonomia characterized by gastrointestinal dysmotility, including intestinal pseudo-obstruction, achalasia, and slow intestinal transit (Dhamija et al., 2008). The putative role of autoantibodies in IBS is substantiated by several lines of evidence, with varying degrees of literature support.

In this review, we aimed to provide a comprehensive overview delineating the existence of various autoantibodies in IBS patients and their underlying functions in pathophysiological research, differential diagnosis, and therapeutic guidance.

## 2 Autoantibodies documented in IBS patients

An Asian study revealed that 18% of IBS patients fulfilling the Rome III criteria were positive for anti-deamidated gliadin peptide IgA antibodies (IgA DGP), and 13% demonstrated a low-grade increase in intraepithelial lymphocytes in duodenal biopsies, and only one of them diagnosed with IBS 2 years ago met the criteria of celiac disease, indicating that a subset of IBS patients had an immunological response to gluten (Lu et al., 2014). Bierła et al. reported that 4.5% of patients with IBS-D were positive for DGP antibodies, 1.6% were positive for anti-tissue transglutaminase 2 (TTG) antibodies, 2.5% were positive for anti-intrinsic factor antibodies, and 12.7% were positive for sIgE against food allergens (Bierła et al., 2023). Zar et al. reported that IgG4 antibodies to foods, such as milk, eggs, wheat, beef, pork, and lamb, were elevated in patients with IBS, and a food-specific IgG4 antibody-guided exclusion diet could improve symptoms (Zar et al., 2005). Dietary modifications can improve IBS symptoms (Rej et al., 2022), but gluten-free diet is not more efficient than low-fermentable oligosaccharides, disacchardies, monosaccharides and polyols (FODMAP)-diet (Dionne et al., 2018), and traditional dietary is recommended as first-choice dietary therapy due to its convenience and minimum cost (Rej et al., 2022).

Following the report of PI-IBS, researchers demonstrated that antibodies against flagellin, a primary structural component of bacterial flagella, were recognized more frequently in patients with IBS than in healthy controls, and these antibodies were found more frequently in PI-IBS than in nonspecific IBS (Schoepfer et al., 2008). Additionally, increased serum levels of lipopolysaccharides and antibodies against flagellin were observed in patients with IBS-D (Dlugosz et al., 2015). This finding suggests that humoral immunity to luminal antigens plays a putative role in the pathogenesis of IBS, especially in patients with PI-IBS. Further studies revealed that antibodies against cytolethal distending toxin B (CdtB, which is the active subunit produced by pathogens and plays a major role in PI-IBS) and vinculin titers were significantly higher in patients with IBS-D than in healthy controls (Rezaie et al., 2017). Antibodies against CdtB can cross-react with vinculin, a protein in the host gut, via molecular mimicry, leading to the development of anti-vinculin autoantibodies (Pimentel et al., 2015a). In the gut, vinculin is present in the myenteric ganglia and in the interstitial cells of Cajal (ICC) which control gut pacing function. Anti-CdtB was negatively correlated with the density of the ICC and myenteric ganglia in a rat model of PI-IBS (Jee et al., 2010). Colocalization results confirmed that anti-CdtB binds to ICC and myenteric ganglia, both in rat models and human subjects (Pimentel et al., 2015a), which may influence the functions of the enteric nervous system (ENS). A Mexican study monitored anti-*Saccharomyces cerevisiae* antibodies (ASCA) in IBS patients and observed that 16.5% of IBS patients were positive for ASCA IgG, however ASCA IgG could not serve as discriminatory biomarker for distinguishing IBS patients for now (Thomas-Dupont et al., 2023).

Antibodies against gonadotropin-releasing hormone (GnRH) and a reduced number of GnRH-containing neurons in the ENS were sporadically found in patients with IBS (Ohlsson et al., 2010), and 11 out of 26 patients with IBS were found to be positive for IgM antibodies to GnRH (Ohlsson et al., 2011). Motawea et al. conducted a meta-analysis and reported a statistically significant association between IBS and increased prevalence of GnRH IgM antibodies (risk ratio = 2.29) (Motawea et al., 2022). In 2012, Wood et al. observed that the positivity rate of anti-enteric neuronal antibodies (AENA) was higher in IBS patients than in non-IBS controls using whole-mount ENS preparations as substrates, which provided substantial evidence of the potential involvement of autoimmunity in ENS (Wood et al., 2012). Using an immunoprotein array test, antibodies in the sera of patients with IBS recognized three antigens: a nondescript ribonucleoprotein (RNP-complex), small nuclear ribonuclear polypeptide A, and Ro-5,200 kDa (Wood et al., 2012). Törnblom et al. screened IBS patients who had ganglionitis with full-thickness jejunal laparoscopic biopsies for common autoantibodies to neural antigens and observed that one IBS patient had antibodies against voltage-gated potassium channels. Another IBS patient had anti-a3-AChR antibodies, which were both against neuronal ion channels (Törnblom et al., 2007). Lütt et al. monitored the levels of anti-neuronal antibodies in gastrointestinal diseases and observed its highest prevalence in IBS, with four of 13 having antibodies against ENS and four against central nervous system neurons (CNS) (Lütt et al., 2018). Our team recruited a large cohort (293 IBS patients) and observed that the AENA-positive rate and obvious positive rate in IBS patients were higher than those in healthy controls (Fan et al., 2018), and 14% of IBS patients had anti-cerebral neuronal antibodies, which were also higher than those in healthy controls, indicating that anti-neuronal antibodies in the sera of IBS patients were mainly targeted to the ENS and a small part of the CNS (Fan et al., 2023).

## 3 Autoantibody-related mechanism underlying IBS pathogenesis

The potential contribution of these autoantibodies to the clinical spectrum of gastrointestinal symptoms remains a noteworthy concern. In recent years, the bidirectional communication of the intestine and brain, as is so called brain-gut-brain interaction or brain-gut axis, has gradually been acknowledged to take part in the pathogenesis of functional bowel disease, including IBS (Niesler et al., 2021). It is difficult to obtain full-thickness biopsy of intestinal tissue including ENS from IBS patients, but there are growing *in-vitro* studies reporting that antibodies produced elsewhere attack ENS, which is commonly referred as the “little brain of gut” due to its diverse neuronal cell types and complexity, via brain-gut axis, producing IBS-like gastrointestinal symptoms. Several studies have demonstrated the pathogenic mechanisms of these autoantibodies, paving the way for illuminating underlying pathophysiology of IBS. Schäfer et al. conducted the earliest study and reported that anti-neuronal antibody-positive sera from PNS patients could increase neuronal death (Schäfer et al., 2000). Initially, incubation of cultured myenteric neurons with anti-HuD-positive sera from patients with paraneoplastic gut dysmotility was thought to induce neuronal apoptosis (De Giorgio et al., 2003). de Giorgio et al. subsequently reported that anti-neuronal antibodies in the sera of patients with chronic pseudo-obstruction may contribute to neuronal dysfunction via autoantibody-mediated activation of autophagy by the Fas receptor (de Giorgio et al., 2008). Li et al. reported that the anti-Hu antibody can directly and falsely activate enteric neurons and visceral sensory nerve fibers, which exert a cytotoxic effect *in vitro* (Li et al., 2016). We previously observed that AENA-positive sera from patients with IBS can promote apoptosis in cultured myenteric neurons of human SH-SY5Y cells and guinea pigs, indicating that AENA-mediated neuropathy may exist in a subset of patients with IBS (Fan et al., 2018).

Notably, a decrease in gastrointestinal motility associated with ENS gliosis and neuronal loss has been observed in a B cell- and antibody-dependent mouse model of multiple sclerosis (Wunsch et al., 2017). Functional studies have revealed that serum anti-neuronal antibodies in patients with gastrointestinal disorders did not influence intestinal motility but could increase epithelial secretion in Ussing chambers (Lütt et al., 2018). These studies indicating that autoantibodies may interplay with the gut-brain axis as part of the pathogenesis of IBS.

## 4 Autoantibodies as biomarkers of IBS

Based on the antibody findings described in the earlier sections, the development of serum biomarkers for IBS from autoimmunity is possible. We have demonstrated the sensitivity and specificity of using AENA to diagnose IBS to be 76.8% and 42.3%, respectively (Fan et al., 2018). Recently, Pimentel et al. demonstrated the benefits of using anti-CdtB and anti-vinculin antibodies as biomarkers to distinguish patients with IBS-D from those with IBD in the workup of diarrhea (Pimentel et al., 2015b). In this clinical trial, the areas under the receiver operating curves were 0.81 and 0.62 for anti-CdtB and anti-vinculin, respectively, for distinguishing IBS-D from IBD. Morales et al. reported that the specificities of anti-CdtB and anti-vinculin for differentiating IBS-D from IBD were 93.5% and 90.9%, with sensitivities of 43.0% and 52.2%, respectively (Morales et al., 2019). Vasapolli et al. demonstrated that both the antibody and gut microbial profiles failed to discriminate between IBS and other functional gastrointestinal disorder subgroups (Vasapolli et al., 2021). We conducted a large cohort study using HuProt™ microarrays and reported that five autoantibodies of IgG and seven IgA were comprehensively involved in differentiating IBS patients from healthy controls with a sensitivity and specificity of 40%–46.7% and 79.4%–86.3%, respectively; however, no specific autoantibodies could serve as serum biomarkers for IBS (Fan et al., 2022).

## 5 Therapeutic intervention based on autoantibody-mediated immune response

Gastrointestinal dysfunction associated with IBS can potentially be reversed by targeting the aforementioned autoantibodies. An IgG-elimination diet combined with probiotics may be beneficial for the management of IBS (Xie et al., 2019). Administration of an anti-IgE monoclonal antibody not only improved asthma but also resulted in almost complete resolution of IBS symptoms (Pearson et al., 2015). Numerous IBS patients perceive that their symptoms are triggered by wheat-containing foods, and one study showed that a gluten-free diet could improve IBS symptoms associated with anti-gliadin IgG and IgA antibodies (Pinto-Sanchez et al., 2021). The infiltration of colonic mast cells and their release of inflammatory mediators in proximity to the mucosal innervation are believed to contribute to the perception of abdominal pain in IBS (Barbara et al., 2004). Previous reports have demonstrated the therapeutic role of IgE blockade with omalizumab (Magen and Chikovani, 2016). Case series have helped identify new therapeutic options, including serum-derived bovine immunoglobulin (SBI), for IBS (Good et al., 2015; Valentin et al., 2017). The action mechanism of SBI is postulated to involve binding to microbial components, maintaining immune balance in the gastrointestinal tract, and managing gut barrier function by increasing the expression of the tight junction proteins zonula occludens-1 and occludin (Petschow et al., 2014).

Anti-neuronal antibodies are capable of inducing neuronal injury and cell death. Studies reported that lipopolysaccharide activation of Toll-like receptor 4 (TLR4) and NF-κB appears to promote the survival of enteric neurons. Factors that regulate TLR4 signaling in neurons may alter gastrointestinal motility (Anitha et al., 2012). 5-HT4 agonists enhance enteric neuronal development and/or survival and decrease the probability of apoptosis (Liu et al., 2009). Thus, TLR4 and 5-HT4 agonists may promote the survival of enteric neurons and mediate neuroprotection and neurogenesis, which may be helpful for neuronal repair. There have been case reports that used systemic steroid therapy to treat patients with ganglionitis manifesting as megacolon and achieved significant clinical improvement (De Giorgio et al., 2002). This immunosuppressive approach warrants further investigation in patients with severe gut motor abnormalities attributable to idiopathic myenteric ganglionitis. Although the pathological mechanisms and outcomes of different autoantibodies vary substantially depending on the diverse target antigens, the underlying neuronal dysfunction reversion by antibody-neutralizing therapies is worth investigating (Höftberger, 2015).

## 6 Conclusion and perspectives

IBS is a globally emergent chronic DGBI exhibiting a complicated pathophysiology and has been a troublesome disease for management thus far. Based on the findings of this mini-review, it can be inferred that a subset of IBS patients exhibit structural alterations, particularly immune activation, including lymphocyte infiltration in the colon and upregulation of proinflammatory cytokines. A full-thickness jejunal biopsy conducted on certain IBS patients revealed indications of inflammatory degenerative neuropathy along with neuronal degeneration. Serum screening of certain IBS patients has confirmed the presence of various autoantibodies, including antibodies produced against certain food allergens and celiac disease-related antibodies; these antibodies might account for the exacerbation of IBS symptoms following the consumption of specific food items. Additionally, antibodies targeting pathogenic subunits, namely, anti-CdtB and anti-vinculin, could bind to the ICC and myenteric ganglia via a molecular mimicry mechanism and affect the functions of the ENS. Anti-neuronal antibodies and antibodies produced against GnRH and neuronal ion channels might affect neuronal dysfunction and intestinal dysmotility. Anti-CdtB and anti-vinculin antibodies can potentially facilitate the differentiation between IBS-D and IBD; however, no specific autoantibodies can serve as exclusive serum biomarkers for IBS. The aforementioned findings present opportunities for the future investigation and illustration of novel therapeutic perspectives for a subset of IBS patients with underlying autoimmune-mediated pathophysiology.
